# Physical Properties of XN (X = B, Al, Ga, In) in the *Pm*−*3n* phase: First-Principles Calculations

**DOI:** 10.3390/ma13061280

**Published:** 2020-03-12

**Authors:** Qidong Zhang, Yucong Zou, Qingyang Fan, Yintang Yang

**Affiliations:** 1 School of Microelectronics, Xidian University, Xi’an 710071, China; qdzhang@xidian.edu.cn (Q.Z.); zouyucong999@163.com (Y.Z.); ytyang@xidian.edu.cn (Y.Y.); 2 College of Information and Control Engineering, Xi’an University of Architecture and Technology, Xi’an 710055, China

**Keywords:** III-V nitride compounds, elastic anisotropy, direct band gap, stability

## Abstract

Three direct semiconductor materials and one indirect semiconductor material, *Pm*−3*n* XN (X = B, Al, Ga, In), are investigated in our work, employing density functional theory (DFT), where the structural properties, stability, elastic properties, elastic anisotropy properties and electronic properties are included. The shear modulus *G* and bulk modulus *B* of *Pm*−3*n* BN are 290 GPa and 244 GPa, respectively, which are slightly less than the values of *B* and *G* for c-BN and *Pnma* BN, while they are larger than those of C_64_ in the *I*4_1_/*amd* phase. The shear modulus of *Pm*−3*n* BN is the greatest, and the shear modulus of C_64_ in the *I*4_1_/*amd* phase is the smallest. The Debye temperatures of BN, AlN, GaN and InN are 1571, 793, 515 and 242 K, respectively, using the elastic modulus formula. AlN has the largest anisotropy in the Young’s modulus, shear modulus, and Poisson‘s ratio; BN has the smallest elastic anisotropy in *G*; and InN has the smallest elastic anisotropy in the Poisson’s ratio. *Pm*−3*n* BN, AlN, GaN and InN have the smallest elastic anisotropy along the (111) direction, and the elastic anisotropy of the *E* in the (100) (010) (001) planes and in the (011) (101) (110) planes is the same. The shear modulus and Poisson’s ratio of BN, AlN, GaN and InN in the *Pm*−3*n* phase in the (001), (010), (100), (111), (101), (110), and (011) planes are the same. In addition, AlN, GaN and InN all have direct band-gaps and can be used as a semiconductor within the HSE06 hybrid functional.

## 1. Introduction

In the 1950s, Germanium was used in low-voltage, low-frequency, medium power transistors and photodetectors in the bright stage. However, there were large short plates in the high-temperature and -radiation resistance of germanium semiconductor devices, so, in the 1960s, germanium gave up its dominant position to silicon. Silicon diffusely serves as a semiconductor material in industry and is mainly used in data computing and other fields. With the increasing demands of science and technology, the defects in the slow transmission speed and single function of silicon have been exposed, so compound semiconductor materials have emerged as required. Third-generation semiconductor materials have many ascendancies, such as a broad band gap, large thermal conductivity, great electron saturation rate, huge breakdown electric field, and a strong ability to resist radiation, so they have a wide range of applications in some blue, green, and violet light-emitting diodes and semiconductor lasers. GaN has a faster speed and higher breakdown voltage compared to silicon. Compared with silicon devices, GaN is more advanced in power conversion efficiency and power density. AlN is an ideal substrate material for advanced high-power light-emitting devices (LEDs, LDs), UV probes and high-power high-frequency electronic equipments. Si and compound semiconductors are two complementary materials. Some of the performance advantages of the compounds make up for the shortcomings of Si crystals, while the production process of Si crystals has obvious irreplaceable advantages, and both of them have certain limitations in the field of application. Therefore, in the application of semiconductors, compatibility means are often used to make the two compatible and utilize their respective advantages. Because of this, more and more attention has been paid to research on new structures and the physical properties of the compounds [1,2,3,4,5,6,7,8,9,10,11,12,13].

Lately, a growing number of studies have been conducted in the field of novel semiconductor materials, such as III-V nitride compound [1,2,3,4,5,6,7,8,9], other III-V compound materials [13,14,15,16,17,18,19,20], carbon-based [14,15,16,17,18,19,20,21], and silicon-based [22,23,24,25,26,27,28]. The structural properties, electronic properties, mechanical attributes, and stableness of the BN polymorph in the *Pnma* structure were investigated, utilizing first-principles calculations by the Cambridge Serial Total Energy Package (CASTEP) plane-wave code, which was studied by Ma et al [1]. They also discovered that *Pnma*-BN has larger band gap (7.18 eV) than other BN polymorphs, and it has an indirect band gap with the HSE06 function [29]. Fan et al. [3] reported the physical properties of AlN, GaN, and InN in the *Pnma* phase. Different from *Pnma*-BN, all the AlN, GaN, and InN materials in the *Pnma* pase have direct band gaps, and *Pnma*-AlN, *Pnma*-GaN, and *Pnma*-InN are the latent binary semiconductor materials for the production of UV detectors, violet photodiodes and infrared detectors, respectively. Compared with the previous materials (AlGaAs, GaAsP, AlGaN, AlGaN and other ternary semiconductor alloys, or AlGaInP quaternary semiconductor alloy), they do not require doping [3]. Liu et al [5] proposed four new AlN polymorphs, named *Pbam*-AlN, *Pbca*-AlN, *Pmn*2_1_-AlN and *Cmcm*-AlN, and Yang et al [6,7] studied the physical properties of six new AlN polymorphs, *Pmn*2_1_-AlN, *Cmcm*-AlN, *Pbca*-AlN, *Pbam*-AlN, bct-AlN (space group: *P*4_2_/*mnm*), and h-AlN (space group: *P*6_3_/*mmc*). All the AlN polymorphs, *Pmn*2_1_-AlN, *Pbam*-AlN, *Pbca*-AlN, *Cmcm*-AlN, *P*4_2_/*mnm*-AlN, are direct band gap semiconductor materials, and their band gaps are 3.63 (*Cmcm*-AlN), 3.89 (*Pmn*2_1_-AlN), 3.86 (*Pbca*-AlN), 3.93 (*Pbam*-AlN), and 5.85 eV (*P*4_2_/*mnm*-AlN), with Perdew–Burke–Ernzerhof (PBE) functionality and PBE0 functionality, respectively. 

In view of the physical properties of the whole III-V nitrides of adamantine phase studied by density functional theory [1,2,3], the band structure of AlN, Gan and InN is direct band gap, which has great potential application in the photoelectric industry or dye-sensitive solar cells [3]. So, according to density functional theory [30,31], the III-V nitride compounds, BN, AlN, GaN, InN in the *Pm*−3*n* phase are first proposed in our paper. The physical attributes of *Pm*−3*n* XN (X = B, Al, Ga, In) semiconductor materials are estimated and discussed, and the stability of *Pm*−3*n* XN (X = B, Al, Ga, In) is also investigated in this work. Additionally, their dynamic and mechanical stability are proven by phonon spectra, elastic constants and related enthalpies. 

## 2. Theoretical Methods

Physical property predictions and structural geometric optimization calculations utilize DFT with ultrasoft pseudopotentials [32] under the Cambridge Sequential Total Energy Package (CASTEP) [33] code in Materials Studio. The exchange correlation potentials are used with the Perdew–Burke–Ernzerhof (PBE) functional of the generalized gradient approximation (GGA) [34], and the Broyden–Fletcher–Goldfarb–Shanno (BFGS) [35] minimization scheme adopts the geometric optimization of the *Pm*−3*n* XN (X = B, Al, Ga, In). A fairly high *k*-point separation (less than or ~ 0.025 Å^−1^ × 2π) is applied to *Pm*−3*n* XN, 10 × 10 × 10, 8 × 8 × 8, 8 × 8 × 8, and 6 × 6 × 6 of the conventional cell for *Pm*−3*n* XN. Additionally, the plane-wave cutoff energy of 420 eV is adopted for structural optimizations and property predictions for *Pm*−3*n* XN (X = B, Al, Ga, In). The Heyd–Scuseria–Ernzerhof (HSE06) hybrid functional [29] was applied to the estimation of the electronic band structures. Finally, all the phonon spectra of *Pm*−3*n* XN adopt the density functional perturbation theory (DFPT) approach [36].

## 3. Results and Discussion

### 3.1. Structural Properties

**Figure 1:**
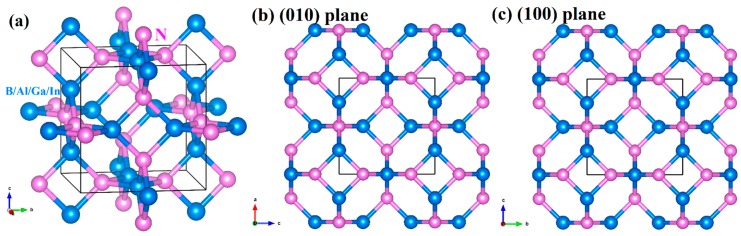
The crystalline structures of *Pm*−3*n* XN (X = B, Al, Ga, In) (**a**), along the (010) direction (**b**) and the (100) direction (**c**).

The crystal textures of *Pm*−3*n* XN (X = B, Al, Ga, In) are illustrated in Figure 1a. Here, red spheres represent nitrogen atoms, blue spheres represent group IIIB elements, and we mainly refer to boron atoms, aluminium atoms, gallium atoms and indium atoms in this work. Figure 1a shows that the quaternary ring structure composed of two nitrogen atoms and two atoms of the group IIIB elements is connected with four vertical quaternary rings. The quaternary ring structure is a square with nitrogen and boron (or aluminium, gallium or indium) atoms at the top of the square. In the conventional cell of *Pm*−3*n* XN, there is a 24-atom cage structure composed of nitrogen atoms and atoms of the group IIIB elements. As both c-BN and *Pm*−3*n* XN belong to the cubic crystal system, the crystal structure of *Pm*−3*n* XN has high symmetry, so the stacking mode of the atoms along the (010) and (100) sides is exactly the same. The related results are plotted in Figure 1b,c. The lattice constants of *Pm*−3*n* XN are presented in Table 1. It can be seen that the lattice constants increase from *Pm*−3*n* BN to *Pm*−3*n* InN. In addition, the lattice constants of c-BN obtained by the GGA method are very close to experimental data, which proves that the lattice constants of *Pm*−3*n* XN obtained in this work are also credible.

### 3.2. Stability

**Figure 2:**
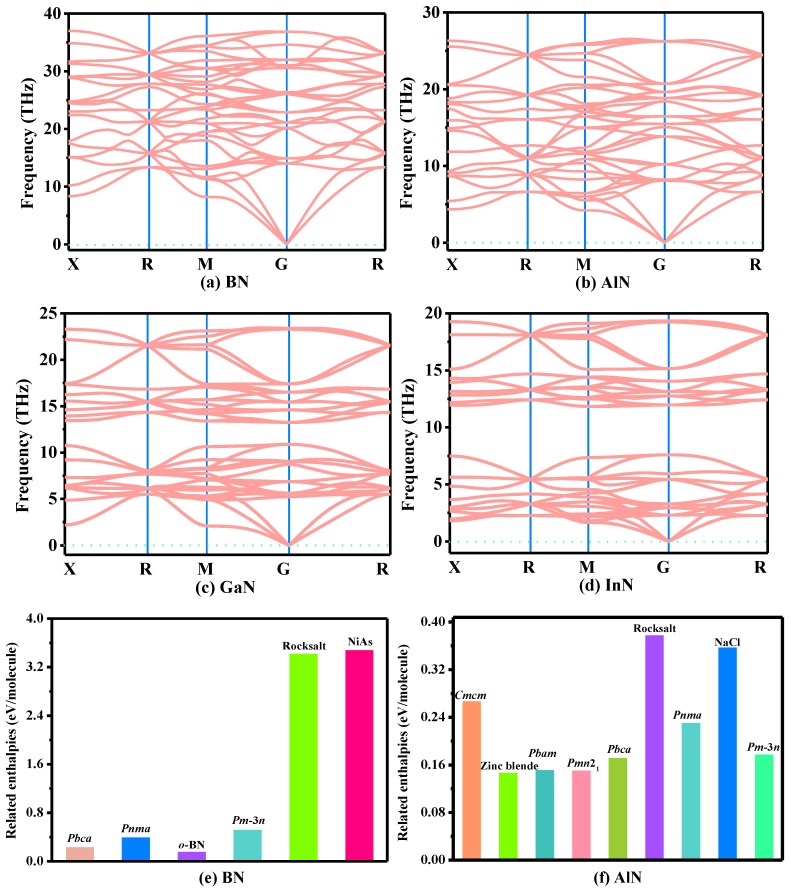

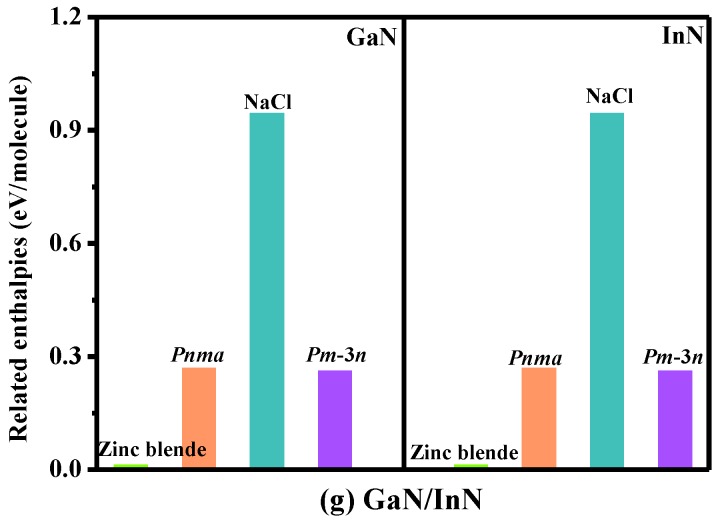
The phonon spectra of BN (**a**), AlN (**b**), GaN (**c**), InN (**d**) in the *Pm*−3*n* phase, and related enthalpies of BN (**e**), AlN (**f**), GaN and InN (**g**).

Stability ploys an extremely significant role in physical performance. The phonon spectra of *Pm*−3*n* XN are plotted in Figure 2a–d. There is no frequency under the zero line, which proves that *Pm*−3*n* XN is dynamically stable. In addition, its mechanical stability is also studied. The highest calculated phonon frequency of the B-N bond-stretching schema in *Pm*−3*n* BN is ~38 THz, which is very close to that of diamond (40 THz), showing that the B-N bond in *Pm*−3*n* BN is relatively strong. The elastic parameters are estimated through the strain stress method, as shown in Table 1. The three necessary and sufficient Born stability criteria for cubic symmetry are taken as: *C*_11_ − *C*_12_ > 0, *C*_11_ + 2*C*_12_ > 0, and *C*_44_ > 0. From Table 1, all the values for *C*_11_, *C*_12_ and *C*_44_ of *Pm*−3*n* XN satisfy the three stability standards for cubic symmetry, which proves that *Pm*−3*n* XN is mechanically stable. Finally, the related enthalpies of *Pm*−3*n* XN are presented in Figure 2e–g. The enthalpy of XN of the wurtzite structure is set to 0. Although the enthalpy of *Pm*−3*n* BN is larger than that of *Pnma* BN [16], it is lower than that of rocksalt-BN and NiAs-BN [19]. For AlN, the enthalpy of the *Pm*−3*n* phase (0.177 eV/molecule) is slightly greater than that of *Pbca* AlN (0.172 eV/molecule) [20], while it is smaller than that of *Pnma* AlN (0.231 eV/molecule) [16]. Similar to *Pm*−3*n* AlN, the enthalpies of *Pm*−3*n* GaN (0.263 eV/molecule) and *Pm*−*3n* InN (0.226 eV/molecule) are also slightly smaller than those of the *Pnma* phase (*Pnma* GaN: 0.271 eV/molecule; *Pnma* InN: 0.237 eV/molecule).

### 3.3. Mechanical and Anisotropy Properties

**Figure 3:**
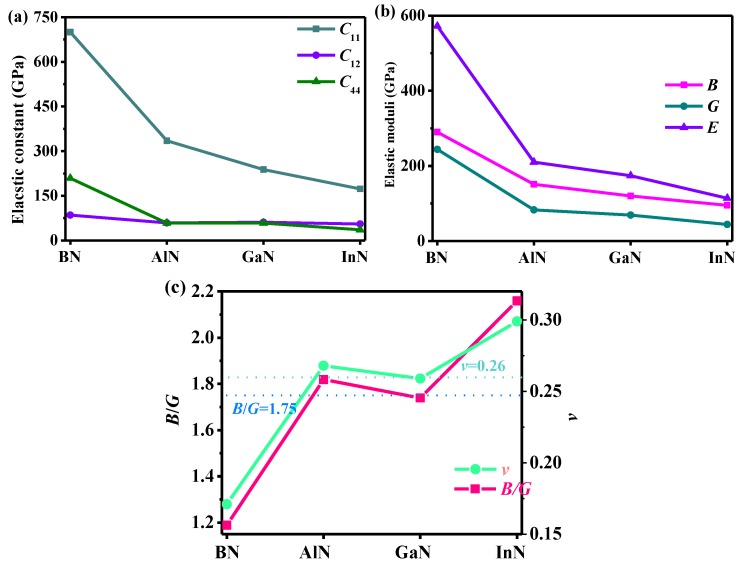
The elastic parameters (**a**) and *B*, *G* and *E* (**b**) of *Pm*−3*n* XN and *B*/*G* and *v* of *Pm*−3*n* XN (**c**).

One can see that the values of *C*_11_, *C*_12_, *C*_44_, *B*, *G*, *E* of the group III-B elements decrease as they change from boron to indium atoms. The *C*_11_, *C*_12_, *C*_44_, *B*, *G*, *E* of *Pm*−3*n* XN are presented in Figure 3. Although the value of *B* for *Pm*−3*n* BN is slightly less than that of c-BN and *Pnma* BN, it is larger than that of C_64_ in the *I*4_1_/*amd* phase. For the shear modulus of *Pnma* BN [16], and C_64_ in the *I*4_1_/*amd* phase [39], the shear modulus of *Pm*−3*n* BN is the largest, and the shear modulus of C_64_ in the *I*4_1_/*amd* phase is the smallest. The *E* and *v* of *Pm*−3*n* XN are given by: *E* = 9*BG*/(3*B* + *G*) and *v* = (3*B* − 2*G*)/[2(3*B* + *G*)] [40,41]. The value of *B* describes the fracture opposition of the material, while the value of *G* describes the plastic deformation opposition of the material, and *E* can be used to describe the tensile elasticity. Therefore, the ratio of *B* and *G* is a significant indicator to evaluate the brittleness or ductility of crystals. In accordance with Pugh [42], a higher *B*/*G* of a solid (more than 1.75) indicates improved ductility, whereas a lower *B*/*G* (less than 1.75) generally indicates brittleness. In addition, the value of *v* is in line with the *B*/*G*, which touches on the ductility of the material; usually, the *v* value is large (*v* > 0.26) [43]. The values of *B*/*G* and *v* for *Pm*−3*n* XN are plotted in Figure 3c. From Figure 3c, *Pm*−3*n* BN is the most brittle, and *Pm*−3*n* InN is the most ductile. *Pm*−3*n* GaN is in between the brittleness and ductility of *Pm*−3*n* BN and *Pm*−3*n* InN, but tends to be brittle.

Knowledge of *Θ*_D_ is a powerful tool to reflect the bonding force between atoms. The value of *Θ*_D_ of different materials is different, and a large melting point indicates that the bond strength of the material is strong, then the Debye temperature increases as the Young’s modulus increases. The Debye temperature can be estimated by the elastic moduli formula. The formula are expressed as [44,45] *v_p_* = [(*B* + 4*G*/3)/*ρ*]^1/2^, *v_s_* = (*G*/*ρ*)^1/2^, and *v_m_* = [(2/*v*${}_{s}^{3}$+ 1/*v*${}_{p}^{3}$)/3]^−1/3^, where *Θ*_D_ = (*h*/*k*_B_)[3*n*/(4π)(*N*_A_*ρ*/*M*)]^1/3^*v_m_*, *B* and *G* typifie the bulk modulus and shear modulus of AlN, GaN and InN, *n* typifies the number of atoms in the molecule, *M* typifies the molecular weight, *ρ* typifies the crystal density, *h* typifies Planck’s constant, *k_B_* represents Boltzmann’s constant, and *N_A_* represents Avogadro’s number. The crystal density, *v*_s_, *v*_p_ and *v*_m_ and Debye temperature of XN in the *Pm*−3*n*, *Pnma* and *F*-43*m* phases are shown in Table 2. For BN in the *Pm*−3*n* and *Pnma* phases, the Debye temperatures are close, while that of BN in the *Pm*−3*n* phase is a little bit larger than that of BN in the *Pnma* phase. Comparing the Young’s modulus, as shown in Table 1, *Pm*−3*n* BN is a little bit higher than that of BN in *Pnma* BN (543 GPa) [16]. For AlN, GaN and InN in the *Pm*−3*n*, *Pnma* and *F*−43*m* phases, there is no doubt that the Debye temperature of XN in the *F*−43*m* phase is the highest, which means that its Young’s modulus is also the largest, the bond strength between the atoms is the largest, that of graphite is the second greatest, and that of cubic diamond is the lowest.

**Figure 4:**
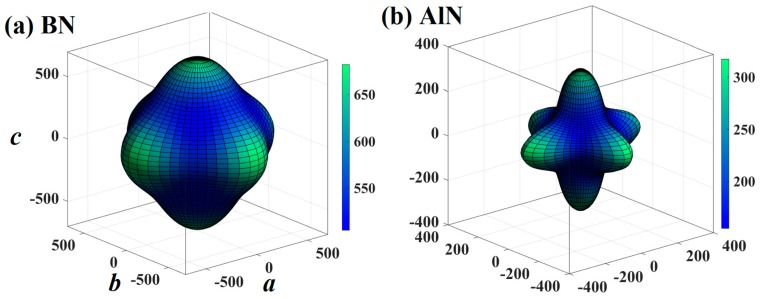

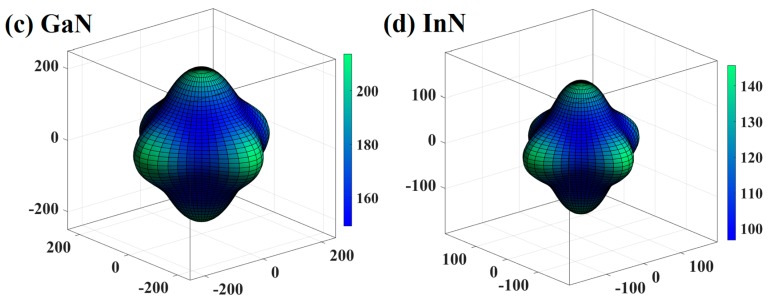
The three-dimensional contour plots of the Young’s modulus for BN (**a**), AlN (**b**), GaN (**c**), and InN (**d**) in the *Pm*−3*n* phase.

**Figure 5:**
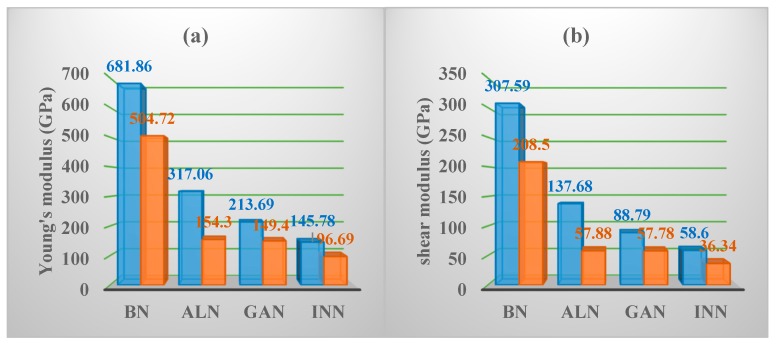

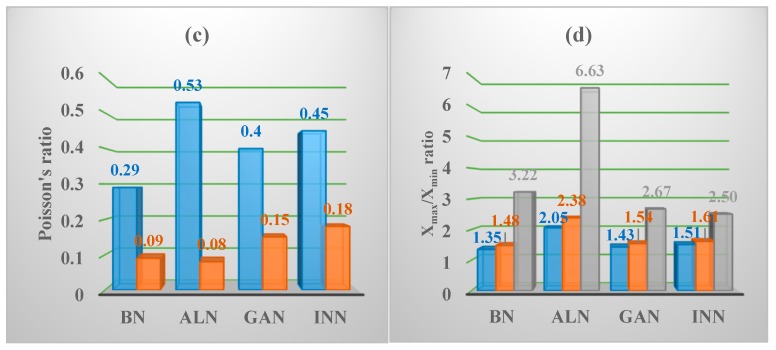
The maximum and the minimum values of the Young’s modulus (**a**), shear modulus (**b**), Poisson’s ratio (**c**), and the *X*_max_/*X*_min_ (**d**) for XN in the *Pm*−3*n* phase.

Mechanical anisotropy is often used to measure in which direction the maximum and minimum values exist. The three-dimensional (3D) graph can show this property intuitively, so the 3D maps of the *E* for BN, AlN, GaN, and InN in the *Pm*−3*n* phase are shown in Figure 4. If the mechanical properties of a material show isotropy in 3D space, the 3D silhouette maps of its mechanical properties are a regular sphere. If the shape is not a sphere, the mechanical properties of the material are anisotropic. At the same time, the less the shape of the 3D graph looks like a sphere, the greater the anisotropy [46]. Therefore, from the three-dimensional contour plots in Figure 4, it can be clearly seen that the mechanical anisotropy of the *E* of AlN is the largest. Similar to other materials [47,48,49,50], the *Y*_max_/*Y*_min_ ratio (where *Y* is *E*, *G* and *v*) is used to quantify the anisotropy of various elastic moduli in this work. The maximum values and the minimum values of the *E* for XN in the *Pm*−3*n* phase are illustrated in Figure 5a, respectively. For Figure 5a, the blue and orange colours represent the *E*_max_ and *E*_min_, respectively. As shown in Figure 3b, the *E*_max_ and *E*_min_ of boron nitride in the *Pm*−3*n* phase are still the largest, while that of indium nitride in the *Pm*−3*n* phase is the lowest. The value of *E*_max_/*E*_min_ for *Pm−3n* BN is shown in Figure 5d, and the blue, orange and grey colours represent the *E*_max_/*E*_min_ ratio, *G*_max_/*G*_min_ ratio, and *v*_max_/*v*_min_ ratios, respectively. It can be concluded from Figure 5d that the *E* of *Pm*−*3n* AlN has the largest anisotropy, as shown in Figure 4. Interestingly, in the *Pnma* phase, the anisotropy of the *E* of AlN is also the greatest [18], while the anisotropy of the *E* of BN in the *Pm*−3*n* phase is also the smallest, and the smallest anisotropy of the *E* in the *Pnma* phase is InN.

To better and more easily comprehend the anisotropy of *E*, and the distribution of E on the major planes of XN in the *Pm*−3*n* phase, such as the (001), (010), (100), (101), (110), (111), and (011) planes, the related *E*_max_ and *E*_min_ of the major planes are listed in Table 3. From Table 3 and Figure 5a, all the maximum values of *E* for XN in the *Pm*−3*n* phase appeared in the (001), (010), (100), (101), (110), and (011) planes, while the minimum values of *E* of XN in the *Pm*−3*n* phase appeared in the (101), (110), and (011) planes. For the (111) plane, both the maximum values and minimum values of *E* of XN in the *Pm*−3*n* phase do not appear in the (111) plane, the *E*_max_ and *E*_min_ for XN in the *Pm*−3*n* phase in the (111) plane are the same, and the *E*_max_ and *E*_min_ are the highest and the lowest values in all directions, which are shown in Figure 5a, so the values of *E* of XN in the *Pm*−3*n* phase in the (111) planes are isotropic. Compared with BN in the *Pnma* phase, the anisotropy of *E* along the (001), (010), (100), and (111) directions of BN in the *Pm*−3*n* phase is also significantly smaller than that in the *Pnma* phase. In addition, for the anisotropy of *E* in the (001), (010), and (100) planes of AlN, GaN and InN in the *Pm*−3*n* and *Pnma* phases, the anisotropy of *E* of the *Pnma* phase is almost the largest, except for the anisotropy of *E* along the (100) direction of AlN in the *Pm*−3*n* phase, which is 1.79, and is slightly larger than that of the *Pnma* phase (1.77 [18]). 

**Figure 6:**
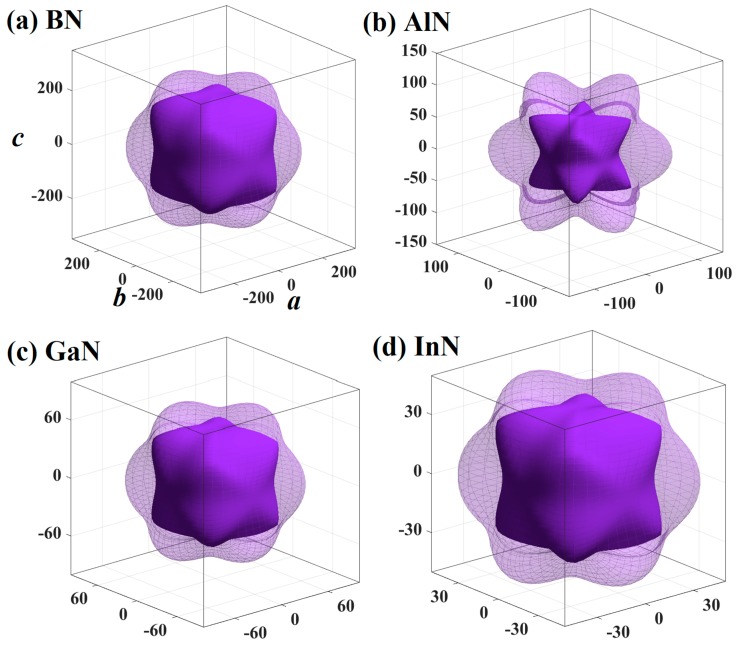
The 3D silhouette maps of the G for BN (**a**), AlN (**b**), GaN (**c**), and InN (**d**) in the *Pm*−3*n* phase.

**Figure 7:**
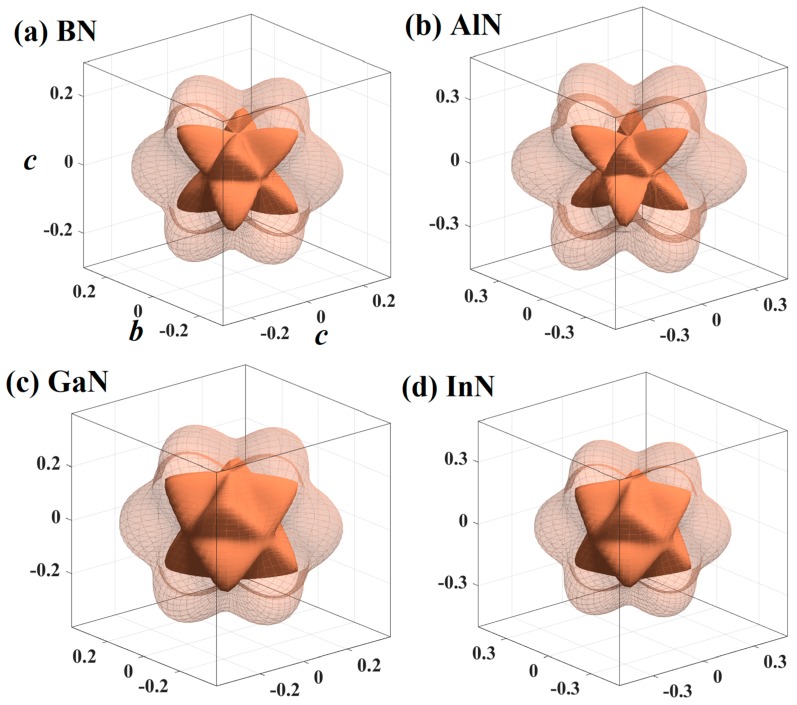
The 3D silhouette maps of the *v* for BN (**a**), AlN (**b**), GaN (**c**), and InN (**d**) in the *Pm*−3*n* phase.

According to Hooke’s law and the ELAM software package [51], we know that *E* can be expressed in two kinds of space angles, while the *G* and *v* need three kinds of space angle. The specific details are described in references [51,52,53]. Therefore, the three-dimensional contour maps of the *G* and *v* are divided into 3D silhouette maps of the maximum values and minimum values. The 3D silhouette maps of the *G*_max_, *G*_min_ *v*_max_ and *v*_min_ for BN, AlN, GaN, and InN in the *Pm*−3*n* phase are plotted in Figure 6a–d and Figure 7a–d, respectively. Here, the dashed purple external and the solid purple external typify the *G*_max_ and *G*_min_, respectively, and the dashed orange external and the solid orange external typify the *v*_max_ and *v*_min_, respectively. The *G*_max_, *G*_min_ *v*_max_ and *v*_min_ and the *X*_max_/*X*_min_ ratio for XN in the *Pm*−3*n* phase are plotted in Figure 5b–d. The blue colours typify the *G*_max_ and *v*_max_, and the orange colours typify the *G*_min_ and *v*_min_ in Figure 5b–c, respectively. The blue, orange and grey colours represent the *E*_max_/*E*_min_ ratio, *G*_max_/*G*_min_ ratio and *v*_max_/*v*_min_ ratio in Figure 5d, respectively. From Figure 6 and Figure 7, it can be seen that the *G* and *v* of XN in the *Pm*−3*n* phase exhibit elastic anisotropy, and from Figure 5b–d, AlN in the *Pm*−3*n* phase has the greatest elastic anisotropy in *G* and *v*, BN in the *Pm*−3*n* phase has the lowest elastic anisotropy in the shear modulus, and *Pm*−3*n* InN has the smallest elastic anisotropy in the Poisson’s ratio. The distribution of *G* and *v* in the (001), (010), (100), (101), (110), (111), and (011) planes of XN in the *Pm*−3*n* phase are also listed in Table 3. Different from the Young’s modulus, all the *G*_max_, *G*_min_ *v*_max_ and *v*_min_ of XN in the *Pm*−3*n* phase appeared in the (001), (010), (100), (101), (110), (111), and (011) planes. In other words, the anisotropy of *G* and *v* of these seven main planes is the same. In addition, for BN in the *Pm*−3*n* and *Pnma* phases, both the elastic anisotropy in *G* and *v* in all directions of the *Pm*−3*n* phase are smaller than that of the *Pnma* phase. Furthermore, both the elastic anisotropy in *G* and *v* along the (001), (010), (100), and (011) directions of the *Pm*−3*n* phase are weaker than that of the *Pnma* phase [16,18]. Especially for *v*, the *v*_max_/*v*_min_ of the *Pm*−3*n* phase is only 3.22, while the *v*_max_/*v*_min_ ratio of the *Pnma* phase is 4.945, and the maximum value is in the (100) plane, which is as high as 14.431. The anisotropy of *G* and *v* of the *Pm*−3*n* phase is obviously smaller than that of the *Pnma* phase.

### 3.4. Electronic Properties

**Figure 8:**
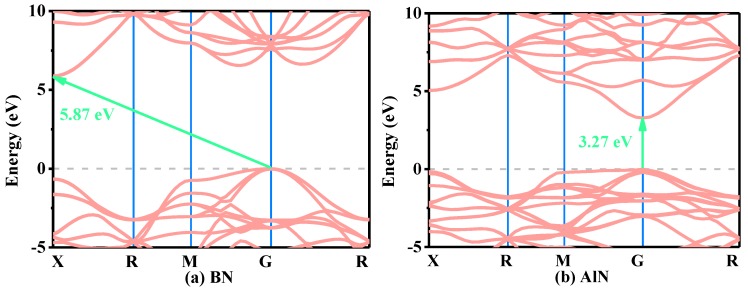

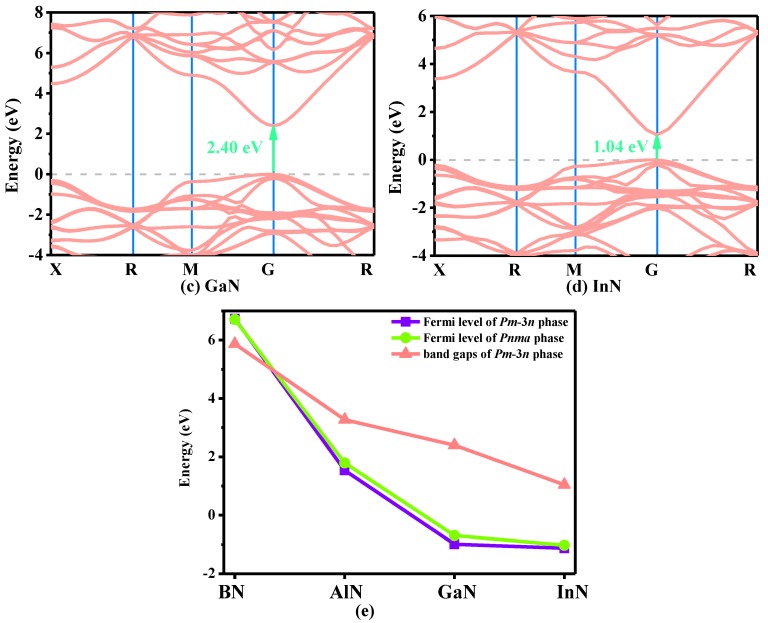
The electronic band structures for BN (**a**), AlN (**b**), GaN (**c**), and InN (**d**), in the *Pm*−3*n* phase, and Fermi level of *Pm*−3*n* and *Pnma* phases (**e**).

The electronic band structures of XN in the Pm-3n phase within the HSE06 function are illustrated in Figure 8a–d. The coordinates of the high symmetry points in the whole Brillouin zone for XN in the *Pm*−3*n* phase are X (0.500, 0.000, 0.000) → R (0.500, 0.500, 0.500) → M (0.500, 0.500, 0.000) → R (0.500, 0.500, 0.500). From Figure 8a–d, AlN, GaN, InN and BN can all be used as a semiconductor material, while AlN, GaN, InN are a direct band gap, and BN is an indirect and broad band gap. Compared with the *Pnma* phase, the band gap of BN in the *Pm*−3*n* phase is slightly less than that of the *Pnma* phase (7.18 eV within the HSE06 hybrid functional) [29]. For AlN, GaN and InN in the *Pm*−3*n* phase and *Pnma* phase with a direct band gap, the band gaps of the same compound in different phases are different. The band gaps of BN, AlN and GaN in the *Pm*−3*n* phase are slightly less than those of BN, AlN and GaN in the *Pnma* phase [16,18], while the band gap of InN in the *Pm−*3*n* phase is 1.04 eV, which is slightly larger than that of the Pnma phase [18]. In addition, the Fermi levels of XN in the *Pm*−3*n* and *Pnma* phases are also estimated in our paper. The related data of XN in the *Pm*−3*n* and *Pnma* phases are shown in Figure 8e. The Fermi levels of XN in the *Pm*−3*n* and *Pnma* phases decrease in turn; the difference is that the reduction degrees of the *Pm*−3*n* phase and *Pnma* phase are different. The Fermi level of XN in the *Pm*−3*n* phase decreases by 7.86 eV, and the reduction degree of XN in the *Pnma* phase is smaller than that of the *Pm*−3*n* phase.

## 4. Conclusions

According to DFT, the structural properties, stability, elastic properties, elastic anisotropy properties and electronic performances of BN, AlN, GaN and InN in the *Pm*−3*n* phase are estimated in our paper. The lattice parameters of InN in the *Pm*−3*n* phase increase by 40.54% compared to those of BN in the *Pm*−3*n* phase, and this increase is larger than that of the lattice parameter a of InN in the *Pnma* phase to BN in the *Pnma* phase (a: 38.59%). This increase is slightly lower than that of lattice parameters b and c of InN in the *Pnma* phase to BN in the Pnma phase (b: 42.87% and c: 41.26%). *Pm*−3*n* BN is the most brittle, and *Pm*−3*n* InN is the most ductile. *Pm*−3*n* GaN is between the brittleness and ductility of *Pm*−3*n* BN and *Pm*−3*n* InN but tends to be brittle. The calculated Debye temperature of XN in *Pm*−3*n* phase follows the order InN < GaN < AlN < BN. By showing the three-dimensional contour plots and the *Y*_max_/*Y*_min_ ratio (where *Y* is shear modulus *G,* Young’s modulus *E*, and Poisson’s ratio *v*), the anisotropy of *E*, *G*, and *v* of the *Pm*−3*n* phase is obviously lower than that of the Pnma phase. The electronic band structures of XN in *Pm*−3*n* phase show that AlN, GaN and InN are semiconductor materials with direct band gaps within the HSE06 hybrid functional, while BN in the *Pm*−3*n* phase is an indirect and wide semiconductor material. Compared with III-V nitrides compounds in *Pnma* phase, the band adjustable range in *Pm*−3*n* phase (1.04–5.87 eV) is slightly smaller than *Pnma* phase (0.66–7.18 eV). It is likely to have a good adhibition in the electronic manufacturing industry such as LEDs, UV detectors, infrared detectors and visible light detectors. Compared with the previous materials (GaAsP, AlGaN, AlGaAs, AlGaN and other ternary semiconductor alloys, or AlGaInP quaternary semiconductor alloy), they save the trouble of making ternary or quaternary semiconductors in semiconductor technology.

## Figures and Tables

**Table 1:** The lattice constants (Å), volumes of the conventional cell (Å^3^), elastic parameters (GPa), *B*, *G*, *E* (GPa) and Poisson’s ratio *v* of *Pm*−3*n* XN and c-BN.

Materials	*a*	*V*	*C*_11_	*C*_12_	*C*_44_	*B*	*G*	*B*/*G*	*E*	*v*
BN	4.438	87.416	700	85	209	290	244	1.189	572	0.171
AlN	5.366	154.505	335	59	58	151	83	1.819	210	0.268
GaN	5.584	174.088	238	61	58	120	69	1.739	174	0.259
InN	6.237	242.570	173	55	36	95	44	2.159	114	0.299
c-BN	3.622	47.517	779	165	446	370	384	0.964	856	0.115
	3.620 ^a^		820 ^b^	190	480	400				

^a^ Reference [37]; ^b^ Reference [38].

**Table 2:** The *v*_s_, *v*_p_, *v*_m_ (m/s), and *Θ*_D_ (K) of XN in the *Pm*−3*n*, *Pnma* and *F*−43*m* phases.

	Space Group	*ρ*	*v*_s_	*v*_p_	*v*_m_	*Θ*_D_
BN	*Pm*−3*n*	2.829	9288	14749	10222	1571
AlN	*Pm*−3*n*	2.643	5604	9950	6235	793
GaN	*Pm*−3*n*	4.793	3794	6651	4217	515
InN	*Pm*−3*n*	5.291	2884	5389	3221	352
BN ^a^	*Pnma*	3.040	8642	14057	9537	1502
AlN ^b^	*Pnma*	2.828	5319	9508	5920	770
GaN ^b^	*Pnma*	5.114	3673	6633	4092	511
InN ^b^	*Pnma*	5.642	2595	5064	2907	325
AlN ^b^	*F*−43*m*	3.206	6169	10488	6837	927
GaN ^b^	*F*−43*m*	5.878	4226	7274	4690	613
InN ^b^	*F*−43*m*	6.496	2962	5493	3307	387

^a^ Reference [16], ^b^ Reference [18].

**Table 3:** The *E*_max_ and *E*_min_ (GPa) and *X*_max_/*X*_min_ in the primary planes for XN in the *Pm*−3*n* phase.

	(100) (010) (001) Plane			(011) (101) (110) Plane			(111) Plane		
	*E*_max_	*E*_min_	*E*_max_/*E*_min_	*E*_max_	*E*_min_	*E*_max_/*E*_min_	*E*_max_	*E*_min_	*E*_max_/*E*_min_
BN	681.86	539.60	1.26	681.86	504.72	1.35	539.60	539.60	1.00
AlN	317.06	177.10	1.79	317.06	154.30	2.05	177.10	177.10	1.00
GaN	213.69	161.49	1.32	213.69	149.40	1.43	161.49	161.49	1.00
InN	145.78	105.53	1.38	145.78	96.69	1.51	105.53	105.53	1.00
	*G*_max_	*G*_min_	*G*_max_/*G*_min_	*G*_max_	*G*_min_	*G*_max_/*G*_min_	*G*_max_	*G*_min_	*G*_max_/*G*_min_
BN	307.59	208.50	1.48	307.59	208.50	1.48	307.59	208.50	1.48
AlN	137.68	57.88	2.38	137.68	57.88	2.38	137.68	57.88	2.38
GaN	88.79	57.78	1.54	88.79	57.78	1.54	88.79	57.78	1.54
InN	58.60	36.34	1.61	58.60	36.34	1.61	58.60	36.34	1.61
	*v*_max_	*v*_min_	*v*_max_/*v*_min_	*v*_max_	*v*_min_	*v*_max_/*v*_min_	*v*_max_	*v*_min_	*v*_max_/*v*_min_
BN	0.29	0.09	3.22	0.29	0.09	3.22	0.29	0.09	3.22
AlN	0.53	0.08	6.63	0.53	0.08	6.63	0.53	0.08	6.63
GaN	0.40	0.15	2.67	0.40	0.15	2.67	0.40	0.15	2.67
InN	0.45	0.18	2.50	0.45	0.18	2.50	0.45	0.18	2.50
